# Workgroup Report: Review of Fish Bioaccumulation Databases Used to Identify Persistent, Bioaccumulative, Toxic Substances

**DOI:** 10.1289/ehp.9424

**Published:** 2006-10-30

**Authors:** Anne V. Weisbrod, Lawrence P. Burkhard, Jon Arnot, Ovanes Mekenyan, Philip H. Howard, Christine Russom, Robert Boethling, Yuki Sakuratani, Theo Traas, Todd Bridges, Charles Lutz, Mark Bonnell, Kent Woodburn, Thomas Parkerton

**Affiliations:** 1 Central Product Safety, The Procter & Gamble Company, Cincinnati, Ohio, USA; 2 National Health & Environmental Effects Laboratory, Office of Research and Development, U.S. Environmental Protection Agency, Duluth, Minnesota, USA; 3 Canadian Environmental Modelling Centre, Trent University, Peterborough, Ontario, Canada; 4 Laboratory of Mathematical Chemistry, Bourgas A. Zlatarov University, Bourgas, Bulgaria; 5 Syracuse Research Corporation, Syracuse, New York, USA; 6 Office of Pollution Prevention & Pesticides, U.S. Environmental Protection Agency, Washington, DC, USA; 7 Chemical Management Center, National Institute of Technology and Evaluation, Tokyo, Japan; 8 National Institute for Public Health and the Environment, Utrecht, the Netherlands; 9 U.S. Army Engineer Research and Development Center, Vicksburg, Mississippi, USA; 10 Environment Canada—New Substances, Ottawa, Ontario, Canada; 11 Toxicology, Environmental Research and Consulting, The Dow Chemical Company, Midland, Michigan, USA; 12 ExxonMobil Biomedical Sciences, Annandale, New Jersey, USA

**Keywords:** BAF, BCF, bioaccumulation, bioconcentration, biota–sediment accumulation factor, BSAF, fish, database, PBT

## Abstract

Chemical management programs strive to protect human health and the environment by accurately identifying persistent, bioaccumulative, toxic substances and restricting their use in commerce. The advance of these programs is challenged by the reality that few empirical data are available for the tens of thousands of commercial substances that require evaluation. Therefore, most preliminary assessments rely on model predictions and data extrapolation. In November 2005, a workshop was held for experts from governments, industry, and academia to examine the availability and quality of *in vivo* fish bioconcentration and bioaccumulation data, and to propose steps to improve its prediction. The workshop focused on fish data because regulatory assessments predominantly focus on the bioconcentration of substances from water into fish, as measured using *in vivo* tests or predicted using computer models. In this article we review of the quantity, features, and public availability of bioconcentration, bioaccumulation, and biota–sediment accumulation data. The workshop revealed that there is significant overlap in the data contained within the various fish bioaccumulation data sources reviewed, and further, that no database contained all of the available fish bioaccumulation data. We believe that a majority of the available bioaccumulation data have been used in the development and testing of quantitative structure–activity relationships and computer models currently in use. Workshop recommendations included the publication of guidance on bioconcentration study quality, the combination of data from various sources to permit better access for modelers and assessors, and the review of chemical domains of existing models to identify areas for expansion.

New national laws linked to enactment of the United Nations Stockholm Convention [Also called “The Persistent Organic Pollutants (POPs) Protocol”] in 2005 have led to significant new activity in the assessment of persistent, bioaccumulative, toxic substances (PBTs) [United Nations Environmental Program (UNEP) 2006]. Canada is assessing approximately 23,000 existing commercial substances on its Domestic Substances List (DSL 1991) for PBT characteristics, and in September 2006 posted a list of categorized substances that may be subject to screening level risk assessment (Canadian Environmental Protection Act 1999). The Registration, Evaluation, and Authorization of Chemicals (REACH) program in the European Union (EU), although not yet fully developed, is likely to expand this effort, as will the integration of PBT evaluation into reviews of new substances in the United States, Japan, Australia, and other nations. One of the largest challenges in preparing PBT assessments is that empirical data are scarce. For example, there are publicly available measured values for 5% (persistent), 4% (bioaccumulative), and 9% (toxic) of the approximately 11,300 organic chemicals being reviewed on the Canadian DSL (Arnot and Gobas 2006; Environment Canada 2006). Due to the general lack of measured data, assessments must rely on several estimation methods or new tests of fish at significant cost. Uncertainty is inherent in PBT assessments, whether the supporting data are measured or estimated, but uncertainty increases when estimation methods are applied to chemical classes for which there are no empirical data.

Notably, measured bioaccumulation data are quite limited compared with the breadth of the chemicals currently used in commerce, as studies have historically focused on the approximately 100 known persistent organic contaminants. For the many chemicals that do not have empirical data, bioaccumulation assessments rely on the extrapolation of data across chemical classes and species, frequently accomplished through the use of quantitative structure–activity relationships (QSARs) and other computer models. The existing models were developed using bioaccumulation data for 60–700 chemicals. By comparing available measured data and model predictions to bioaccumulation criteria in Canada [i.e., bioconcentration factor (BCF) or bioaccumulation factor (BAF) ≥ 5,000], approximately 1,240 organic chemicals on the DSL were identified as potentially bioaccumulative (Environment Canada 2006; Persistence and Bioaccumulation Regulations 2000). On the basis of a pilot exercise, it was estimated that simply collecting the existing environmental toxicity and fate data on 1,240 chemicals requires 82 work-years of effort (Weisbrod AV, personal communication). Further, regulatory guidelines indicate mandatory testing. The only standard test is the Organization for Economic Cooperation and Development (OECD) Technical Guideline (TG) 305, which costs approximately $125,000/chemical (Woodburn and Springer 2004). The need for more empirical data to better understand the bioaccumulative characteristic of chemicals is being counterbalanced by the desire from various groups, such as animal welfare organizations, to reduce or eliminate vertebrate testing, including testing in fish.

Given the current level of regulatory interest in PBT assessments, the International Life Sciences Institute (ILSI) Health and Environmental Sciences Institute (HESI) agreed to organize a multiple stakeholder committee of experts and coordinate a series of international workshops to gain scientific understanding and consensus on the advancement of bioaccumulation assessment techniques, ultimately to reduce risk to the environment and humans by providing effective and consistent methods for assessing the bioaccumulation potential of commercial chemicals. In April 2005, the first workshop on this topic was sponsored by ILSI-HESI and Procter & Gamble, and brought together experts in *in vitro* and *in vivo* testing, QSARs, extrapolation, bioaccumulation modeling, and testing. Techniques and methods were discussed for improving bioaccumulation assessments [e.g., reducing animal testing, tiered approaches for bioaccumulation assessment, potential revision of the existing *in vivo* OECD TG 305 bioconcentration test (OECD 1996)], and how new methods could be efficiently developed in time to support the Canadian and the EU agencies in their evaluation of potential PBTs. One outcome of the workshop was partnerships to develop *in vitro* assays, provide new data on representative chemicals, and communicate how tiers of information from models and *in vitro* and *in vivo* tests can be used to build a weight of evidence regarding a chemical’s bioaccumulation potential. The other outcome was a plan for several workshops.

One of these workshops, the In Vivo Bioaccumulation Database Workshop, focused on *in vivo* fish bioaccumulation databases and data quality evaluation methods, and was held in conjunction with the North America Annual Meeting of the Society of Environmental Toxicology & Chemistry (SETAC) 11–12 November 2005 in Baltimore, Maryland, USA. The next workshop, the *In Vitro* ADME Workshop, focused on the use of *in vitro* absorption, distribution, metabolism, and excretion (ADME) tests for bioaccumulation assessment, and was held in conjunction with the Society of Toxicology Annual Meeting 3–4 March 2006 in San Diego, California, USA. The last workshop, sponsored by the HESI, the European Chemicals Bureau, and SETAC-Europe, was held in conjunction with the SETAC-Europe Annual Meeting, 5–6 May 2006 in the Hague, Netherlands. This workshop focused on integrated testing strategies for bioaccumulation in the POPs Protocol and the REACH Implementation Projects 3.2 and 3.3.

The focus of the November 2005 workshop on fish *in vivo* bioaccumulation databases was to explore the sources and state of empirical fish bioaccumulation data. In this report from the workshop, we provide an overview of the main sources of *in vivo* bioaccumulation data, as well as the different computer models used in the chemical management programs that incorporate them. A second workshop report (Parkerton T, Woodburn KB, Arnot JA, Weisbrod AV, unpublished data) will provide guidance on how to assess the quality of BCF test data. The goal of these reports is to assist in making reliable *in vivo* fish bioconcentration and bioaccumulation data more accessible for chemical assessment and model improvement.

## Definitions

The terms “bioconcentration” and “bioaccumulation” are both used in assessment of the hazard and risk of chemical contamination in the environment, according to various regulatory criteria [European Commission 2003; U.S. Environmental Protection Agency (EPA) 2000; Persistence and Bioaccumulation Regulations 2000]. Bioconcentration is the process by which a chemical is retained in an aquatic organism following its absorption through respiratory and dermal surfaces from the surrounding water (does not include dietary exposure). Bioconcentration is measured under controlled laboratory conditions (OECD 1996). The potential for a chemical to bioconcentrate is expressed by its BCF, which may be calculated in two ways: *a*) using the ratio of the chemical concentration in the organism *C*_B_ and the concentration in the water (*C*_W_) at steady state, so *BCF*_SteadyState_ = *C*_B_/*C*_W_; or *b*) using the ratio of the rate of chemical uptake (*k*_1_) and the total rate of chemical elimination or depuration (*k*_2_) in the organism, so *BCF*_Kinetic_ = *k*_1_/*k*_2_ (Barron 1990; Mackay and Fraser 2000).

Bioaccumulation is net uptake and retention of a chemical in an organism from all routes of exposure (diet, dermal, respiratory) and any source (water, sediment, food) as typically occurs in the natural environment (Spacie et al. 1995). Bioaccumulation by invertebrates can be measured in the laboratory (ASTM 2000) or in the field. For vertebrates, bioaccumulation is measured in the field and expressed using either a BAF, the ratio of chemical concentration in the organism to the chemical’s concentration in water, or a biota–sediment accumulation factor (BSAF; the ratio of lipid-normalized chemical concentration in the organism to that in the sediment on an organic carbon basis) (Spacie et al. 1995).

## Data Sources

The main sources of bioconcentration, bioaccumulation, and biota–sediment accumulation data for fish are shown in Table 1 and described in detail below.

### ECOTOX database

The U.S. EPA’s ECOTOX (ECOTOXicology) database (U.S. EPA 2006a) is the largest compilation of eco-toxicity data, providing information on the effects of single chemical stressors to aquatic and terrestrial species. The source of ECOTOX data are published studies, primarily from the peer-reviewed literature, and third-party electronic data files submitted by U.S. and international government agencies. As of November 2005, the ECOTOX database included > 480,000 test records, summarizing data from > 6,000 aquatic and terrestrial species and 10,000 chemicals. The database is updated with new test records on a quarterly basis. Ten major effect groups are included in ECOTOX: behavioral, biochemical, cellular, growth, mortality, physiology, population, reproduction, ecosystem, and accumulation. The accumulation subfile contains > 15,000 BCF and BAF values, gleaned from > 1,100 publications and encompassing approximately 700 distinct chemicals. The majority of the accumulation results are for aquatic species (94%), but data are also available for terrestrial species. The use of the online database is free and is available online (U.S. EPA 2006a).

The ECOTOX website (U.S. EPA 2006a) contains a search engine for parameter searching and data downloading. Data can be queried by chemical name, CAS Registry number, organic chemical class, or species name (scientific and common) from laboratory and field data in either fresh or saltwater. Pertinent information on species, chemicals, test methods, and test results are codified, including alkalinity, chemical concentration in test solutions, chemical analysis method, type(s) of control treatments, duration, exposure type, experimental design, organic carbon content (e.g., in sediment or particulate water fraction), temperature, tissue analyzed, water type, pH, and source reference. Data are available for downloading as comma delimited ASCII data files. ECOTOX does not include an assessment of each study’s acceptability or quality. It is recommended that data obtained be verified against the original sources.

### Japan METI-NITE Database

Chemicals are tested in Japan according to the Chemical Substances Control Law (CSCL). A bio-concentration test is required for chemicals that are considered to be not readily biodegradable according to the CSCL. Bioconcentration tests are conducted based on the OECD TG 305C method (OECD 1996). Approved laboratories conduct all studies in compliance with Good Laboratory Practice (GLP), and study reports are reviewed by the joint council of three ministries [Ministry of Economy, Trade and Industry (METI); Ministry of Health, Labour and Welfare (MHLW); and Ministry of the Environment (MoE)]. Measured BCF data exist for 3,100 “new” chemicals and 800 “existing” chemicals, with approximately 300 new chemicals being tested every year. Data on new chemicals are proprietary and not available to the public; data on existing chemicals are publicly available online [National Institute of Technology and Evaluation (NITE) 2006].

NITE performs an initial evaluation of submitted data for the joint ministries and manages the database. The data are also used for generating the criteria for development of QSARs and validating QSAR models. The BCF data for about 800 existing chemicals are available at the Chemical Risk Information Platform of NITE’s web site (NITE 2006). In this system, biodegradation and bioconcentration test results can be searched using substance name, CAS Registry number, test method, and test condition. Available information includes chemical structure, CAS Registry number, test conditions, measured biodegradability, and BCF value. Maximum and minimum BCFs at two different exposure concentrations are reported for the standard test species, the common carp (*Cyprinus carpio*). The duration of exposure and exposure method (usually flow through) and lipid content are usually provided, and occasionally the analytical method (e.g., gas chromatography with electron capture detection) is included in the database.

### The National Library of Medicine’s Hazardous Substances Data Bank

The Hazardous Substances Data Bank (HSDB) is a toxicology data file on the National Library of Medicine’s (NLM) Toxicology Data Network (TOXNET; NLM 2006). The HSDB consists of > 4,800 chemical records. The data are fully referenced and peer-reviewed by a scientific review panel composed of expert toxicologists and other scientists. The database can be searched by chemical structure, chemical name, chemical name fragment, CAS Registry number, or subject terms. Data may be selected from areas such as human health effects, emergency medical treatment, animal toxicity studies, metabolism/pharmacokinetics, pharmacology, environmental fate/exposure, and chemical/physical properties. The information available at this site may be viewed, printed, or downloaded. Although the data are associated with primary source references, there is little information presented on experimental details aside from the reported BCF values. The HSDB is accessible, free of charge, via TOXNET (NLM 2006).

### Environmental Fate Data Base

In November 1979, the Syracuse Research Corporation (SRC) initiated the Environmental Fate Data Base (EFDB) under the sponsorship of the U.S. EPA. This computerized database (SRC 2006) is composed of several interrelated files: DATALOG, CHEMFATE, BIOLOG, and BIODEG. DATALOG, the largest file, contains > 325,000 records on > 16,000 chemicals derived from the literature (Meylan et al.1999). Categories of data include chemical identification and property information [e.g., chemical name, CAS Registry number, molecular formula, molecular weight, octanol–water partition coefficients (*K*_ow_), and acid dissociation constant]. Each record also includes a reference to the data source and a summary of experimental design, methods, and results. This database contains some bioaccumulation and bioconcentration information; however, data exist only for a small fraction of the chemicals in the database. The database does not differentiate between BCF values that were derived experimentally or from models. The database can be obtained from the author or freely accessed online (SRC 2006).

### SRC Fish Bioconcentration Database

The SRC also has a database of fish experimental BCF values for 694 chemicals. The data were acquired from ECOTOX, HSDB, EFDB, and METI-NITE databases. The database was constructed using ISIS/Base software (Elsevier-MDL, San Ramon, CA, USA) to enable searches by molecular substructure (ISIS/Base software is required to use the database). This database includes the BCF (a maximum of 10 values per chemical), measured chemical concentration in water, exposure duration, species and tissue used for analysis, method of calculating the BCF, and whole fish or tissue-specific lipid content when available. The primary reference source is also presented for each chemical. These data have not been assessed for quality (Howard PH, personal communication). The database can be obtained from SRC.

### RIVM Database

The Dutch National Institute for Public Health and the Environment (RIVM) e-toxBase is a database containing aquatic toxicity, terrestrial toxicity, and BCF data. The database was launched in 2003, and new interfaces were provided in 2004. The database has > 160,000 records of persistent, bioaccumulative, and toxic substances data. The data were obtained from the U.S. EPA ECOTOX database (U.S. EPA 2006a) and from past and ongoing chemical evaluations in the EU. Records within the database can be flagged as confidential and have access limited to defined user groups. The data are not evaluated for quality or reliability, but the database stores all remarks and information from quality assessments performed by the users of the data, on an individual compound basis. Access to the database is currently limited to RIVM staff.

#### Handbook of Physical–Chemical Properties and Environmental Fate for Organic Chemicals

The *Handbook of Physical–Chemical Properties and Environmental Fate for Organic Chemicals* (Mackay et al. 2006) consists of a number of volumes, each covering a set of related organic chemical substances. The handbook is available in book and CD-ROM format. This handbook includes BCF, *K*_ow_, and other physical chemical property values relevant for environmental fate assessments.

### Comparative QSAR

The book Comparative QSAR has a dataset of 802 experimental BCF values for 209 chemicals (Devillers 1998). The book includes summaries for each value from the original publications. These summaries include organism information (e.g., species, sex, weight, lipid content, and size), test conditions and methods as well as the method for deriving the BCF value (e.g. kinetic or steady state).

### Environment Canada BCF and BAF Database

The Environment Canada BCF and BAF database contains empirical values for organic chemicals reported for 219 non-mammalian aquatic species, primarily fish. The database exists in a Microsoft Excel spreadsheet (Microsoft, Redmond, WA, USA). Fields and parameters include the test chemical, biological, exposure, and end point calculation information, as well as the primary source reference. The database includes approximately 5,300 BCF and 1,650 BAF values for about 830 and 120 chemicals, respectively. Laboratory feeding studies were not included in the database. The data were acquired by reviewing reference sources from various databases (e.g., ECOTOX, HSDB, EFDB, SRC, and METI-NITE) and from key word searches of the published scientific literature. Primary source literature was consulted to confirm database values, to remove repeated “overlapping” values from the compilation, and to obtain key information for evaluating the general quality of the empirical values. Data were assessed for quality according to a set of criteria based on the OECD TG 305E bioconcentration test protocol (OECD 1996). The database is available by personal request from Environment Canada-Existing Substances (Ottawa, Ontario, Canada).

### CONCAWE Database

CONCAWE (Conservation of Clean Air and Water in Europe), the oil companies’ European Association for Environmental, Health and Safety in Refining and Distribution, contracted with the SRC to develop a database of experimental BCF values in fish for hydrocarbons (i.e., chemicals containing only hydrogen and carbon) (Stewart et al. 2005). The database was assembled by abstracting all hydrocarbon data from the SRC fish bioconcentration database (73 chemicals) and performing a comprehensive literature search. This search resulted in 16 new compounds (3 of which contained sulfur), and covered DATALOG, EFDB, HSDB, ECOTOX, TOXLINE, BIOSIS, and SCISEARCH databases. All data were subsequently reviewed for quality; only BCF values determined using whole body analysis, where the parent chemical was directly measured, were included for BCF tests with radiolabeled and non-radiolabeled parent chemical. The data evaluation resulted in 7 chemicals being removed from the original list of 73 chemicals. The final database contains BCF values for 84 hydrocarbons. The database is similar to the SRC fish bioconcentration database in format but includes a field for describing the analytical method. This database is currently unavailable to the general public, although more than half of the data are available as part of the SRC fish bioconcentration database.

### U.S. Army Corps of Engineers, Engineer Research and Development Center BSAF Database

The Engineer Research and Development Center (ERDC) BSAF database (U.S. Army Corps of Engineers ERDC 2006) currently contains 2,135 records for 237 species and 205 chemicals, obtained from 183 references. Most of the data is extracted from the peer-reviewed literature, and includes BSAFs derived from field and laboratory studies but not from theoretical calculations. The BSAFs are not reviewed for data quality. The database includes the BSAFs, their variances, and the number of replicates. The BSAF database is publicly accessible online (U.S. Army Corps of Engineers ERDC 2006) and contains BSAFs for nonpolar organics, organo-tin compounds, and organism lipid values. The database is updated at irregular intervals and is readily searchable by species and/or chemical name. The ability to search by CAS Registry number is planned for a future upgrade.

### U.S. EPA BSAF data set

The U.S. EPA Office of Research and Development is developing a dataset of BSAF values from Superfund remedial investigations. The BSAF data are from field measurements performed at Superfund sites, and a diverse group of chemicals is covered, including polychlorinated biphenyls, polychlorinated dibenzo-*p*-dioxins, polychlorinated dibenzofurans, polycyclic aromatic hydrocarbons, and chlorinated pesticides. The data are packaged in a Microsoft Access database file and are search-able by chemical, species, and site or subsite. Completion of the database is projected for late 2006. Database fields include information on sediment and biota samples (e.g., collection location and dates; sediment depth analyzed; average concentrations, variances, and number of samples for sediment and biota; organism lipid and sediment organic carbon content). The dataset will be freely available to the public in 2007 (Burkhard LP, personal communication).

## Quality Assessment of Coded Data

The “quality” of a database is related to the quality of the data, but is more correctly related to the measures taken to ensure the integrity and correctness of data entered into the system; this is what makes up a quality database. A quality data set may differ depending on its application. Data used in a QSAR model require a different level of quality or conformity than data used in screening level risk assessments. Because of the effort and cost involved with maintaining a database (e.g., adding new entries to keep the database up to date and maintaining software), most providers of bioaccumulation data post their collections of data with assessments of the integrity and correctness of the data, but not assessments of the data reliability for different applications. An additional layer of assessment for data reliability would, in all likelihood, reduce the amount of data available for a particular model application.

Unknown data quality (primarily data reliability) was identified as a key limitation to many existing databases, in part because most bioaccumulation databases do not include sufficient details of what the data represent from the study (e.g., the whole body accumulation of radiolabeled test substance and metabolites, the accumulation of parent compound only in specific tissue). Consequently, retrieved bioaccumulation data for an individual compound sometimes vary widely (up to a few orders of magnitude), and users of the data cannot determine why such variability exists without examining the original source of measured data. Causes for the broad ranges include inconsistencies in the testing protocol, different reporting formats among studies, improper testing technique (e.g., exceeding the aqueous solubility of the chemical in the test), and the comparison of BCF data across taxonomic orders with different metabolic abilities (e.g., bivalves vs. fish). Because measured bioaccumulation data are scarce, there is a tendency to use or group the available data, without assessing whether the result combines “apples and oranges.” Attempts to evaluate data quality have also been limited by the lack of a standard guideline that identifies key criteria to be evaluated, or a systematic method to select data representing the same observation (e.g., measured concentration of parent compound in a whole fish with intact skin and gastrointestinal tract).

Currently, it is unclear how statistical error, uncertainty, and variability are captured and expressed within the bioaccumulation databases described above. Similarly, data from older studies that do not meet all requirements of the OECD TG 305 BCF test (OECD 1996) may still be valuable, if the differences are appropriately identified and constrained. Users of bioaccumulation data need to be aware of the statistical error, uncertainty, and variability associated with bioaccumulation data retrieved from databases and their impact when used in direct applications, as well as in the development of predictive models. As mentioned above, this article’s companion workgroup report (Parkerton T, Woodburn KB, Arnot JA, Weisbrod AV, unpublished data) will provide the technical guidance on how bioaccumulation data should be evaluated and give examples of how such considerations can be practically applied in assigning data use or accuracy categories to *in vivo* bioaccumulation studies.

In brief, workshop participants recommended that a reliability evaluation be incorporated into future database development, because the value of any database will be determined by the quality of the data it contains. Participants suggested that a systematic approach similar to a Klimisch scale (Klimisch et al. 1997) should be used to evaluate data accuracy and precision. This data evaluation scheme has been adopted in the International Uniform Chemical Information Database (IUCLID) and the OECD Screening Information Data Set (SIDS) program, and is conceptually similar to the approach used by the U.S. EPA in the High Production Volume Challenge (HPV) Program. The decision to place a study into a specific category depends on a number of key considerations including unambiguous test substance identity, appropriate analytical determination of the test substance in the exposure medium and organism tissue, suitable exposure conditions during the uptake phase of the test, adequate test organism health over the study period, and a clearly defined bioaccumulation test end point that reflects a steady-state situation.

## Bioaccumulation Assessment Approaches

Environment Canada uses a weight-of-evidence approach to determine bioaccumulation potential for their categorization and screening of the DSL. Their decisions incorporate experimental data for the chemical of interest, or data extrapolated across chemical classes and species when available. In the absence of reliable empirical BCF or BAF data for organic chemicals with a log *K*_ow_ > 4.1, predictions from three BCF models and one BAF model are used to assess bioaccumulation. The models used are the SRC BCFWIN QSAR model (Meylan et al. 1999), the Mekenyan-Dimitrov POPs model (Dimitrov et al. 2005a), and the Gobas-Arnot BCF and BAF models (Arnot and Gobas 2003).

The U.S. EPA, under its Pre-Manufacture Notification process, uses the SRC BCFWIN model when no measured data are available (U.S. EPA 2003). BCFWIN is incorporated into the PBT Profiler model suite, which is available for public use (U.S. EPA 2006b). For the development of water quality criteria, the U.S. EPA uses a tiered approach (in decreasing order of preference) consisting of *a*) measured BAF values; *b*) BAF values estimated from measured BSAF values, and estimated or measured sediment–water column chemical concentration quotient data; *c*) BAF values estimated from measured BCFs and food chain multipliers; and *d*) BAF values estimated using *K*_ow_ and food chain multipliers. Food chain multipliers were determined using the Gobas food web model with national average conditions and parameters (U.S. EPA 2000).

In Japan, the METI performs bioaccumulation assessments for new and existing chemicals. Under their Chemical Substance Control Law (CSCL), chemicals are subjected to the OECD TG 301C Biodegradation Test (OECD 1992). If the parent residue is ≤ 40% and the metabolite residuals are < 1% (relative to the initial parent residue in the biodegradation test, then the chemical is considered readily biodegradable and a bioaccumulation assessment and testing is not required. For chemicals that are not judged to be readily biodegradable, METI requires the OECD TG 305C Bioconcentration Test to be performed. For chemicals with log *K*_ow_ values < 3.5 or molecular weights > 800, bioconcentration testing is not required because these chemicals are not considered to be bioaccumulative. The METI, with support from NITE, uses linear log BCF–log *K*_ow_ relationships, developed from the NITE database of BCF data, for screening and prioritization of untested existing chemicals.

Until 2007, the EU assesses the bioconcentration potential of chemicals using laboratory-measured or predicted BCF values using QSAR equations (Veith et al. 1979). In most cases, preference is given to BCF values measured using the OCED 305E guideline (OECD 1996), and BCF measurements have been mandatory for the registration of new chemicals with anticipated use volumes of > 100 metric tons/year/manufacturer. However, the EU methodology for assessing bioconcentration potential is changing because of their new chemical management program, REACH, expected to be implemented in 2007. The types of bioconcentration data required and optional for REACH will soon be defined; this was the subject for the Joint HESI/European Chemicals Bureau/SETAC-Europe Workshop.

## Models for Predicting Bioconcentration and Bioaccumulation

### Bioconcentration models

In 1974, the first relationship based upon a chemical’s *K*_ow_ was established for predicting BCFs of nonionic organic chemicals (Neely et al. 1974). This relationship was of the general form


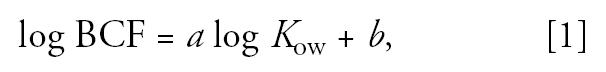


where *a* and *b* are empirical constants derived by regression analysis of BCF-*K*_ow_ data sets. Since then, numerous regression equations have been developed with varying amounts of bioconcentration data (Bintein et al. 1993; Schüürmann and Klein 1988; Veith et al. 1979). Based on the analyses of BCF data and underlying partitioning theory based on *K*_ow_ (deWolf et al. 1992), the slope of the regression equation should be close to 1 and the intercept should be approximately zero. These regressions apply for BCF-*K*_ow_ data sets of organic chemicals that *a*) they are nonionic, *b*) they have small molecular weight (< 1,000 g/mol), *c*) they are metabolized very slowly or not metabolized, and *d*) when BCF values are expressed by the chemical concentration in whole fish, the chemicals are normalized to their lipid content and the bioavailable (or freely dissolved) concentration of the chemical in water.

#### BCFWIN

BCFWIN is a QSAR model contained within the Estimation Programs Interface (EPI) Suite; it was developed by the U.S. EPA Office of Pollution Prevention and Toxics and the SRC. The EPI Suite contains 11 programs for estimating physical–chemical properties, rate constants, and partition coefficients for organic chemicals; one of these programs is BCFWIN, which is used to estimate the chemical’s BCF based on its *K*_ow_ and structural features (e.g., functional groups and elemental composition) (Meylan et al. 1999). The BCFWIN predictive algorithm is built on a database of 694 chemicals that includes 610 nonionic organic compounds (which include 18 organometallics) and 84 ionic organic compounds (which include carboxylic acids, sulfonic acids and their salts, and quaternary nitrogen compounds). BCFWIN is publicly available online (U.S. EPA 2006b) and is also integrated into the PBT Profiler models used to assess bioaccumulation.

The BCFWIN predictive model is a refinement of the regression equation approach presented by Neely et al. (1974), with a much larger database of BCF values that permit the development of correction factors for specific chemical classes and molecular structures. The model reasonably predicts BCF values for chemicals within the model’s domain of applicability. Based on comparison of estimated and measured BCFs in the BCFWIN training set (i.e., 694 chemicals), 50%, 82%, and 90% of the estimated log BCFs are one-half, three-quarters, and one log unit of their measured values, respectively, and have the correlation coefficient (*r*^2^) of 0.73.

As discussed above, the BCFWIN database assembly process did not evaluate the quality of individual studies incorporated into the database. However, rules were developed for assigning a chemical’s recommended BCF value from the list of reported values assembled, and these assignments were made for the 694 chemicals. These rules including selecting kinetic studies for chemicals with low solubility, equilibrium studies that were run the longest to allow for equilibrium to be achieved, and studies that had the lowest test chemical concentration to avoid toxicity. Therefore, for chemicals with more than one BCF value, a weight-of-evidence approach was used. As for any model, if all the studies for a chemical had poorly designed protocols, these uncertainties would be carried in the recommended value. Any uncertainties incorporated into the list of 694 selected BCF values are directly translated into the predictive model. Uncertainties also arise from the quality of the *K*_ow_ data for individual chemicals used in the BCF-*K*_ow_ training set. Most of the *K*_ow_ values in BCFWIN were measured values from a high quality database, and the estimated *K*_ow_ values used a method with a mean error of about 0.30 log units.

#### CONCAWE

The algorithms used by the BCFWIN program were extended to hydrocarbons by developing a correction factor for the hydrocarbon chemical class (Stewart et al. 2005). The hydrocarbon correction factor was developed using the new set of recommended BCF values for 84 hydrocarbons. For the hydrocarbons, the mean absolute error and SD for the predicted log BCF values were 0.43 ± 0.54, and the correlation coefficient (*r*^2^) between measured and predicted log BCF values was 0.60.

#### Baseline model (POPs)

This bioaccumulation model is part of the OASIS model suite developed by the Laboratory of Mathematical Chemistry at Bourgas University (Bourgas, Bulgaria) and used by new chemical agencies in several countries. Demonstration versions are available online (Laboratory of Mathematical Chemistry 2006). The baseline concept for modeling the bioconcentration of chemicals is based on a reference curve delineating the empirically observed maximum bioconcentration driven by hydrophobicity of chemicals (Dimitrov et al. 2005a). In fact, this is the highest log *BCF* (log *BCF_max_*) that could be reached for a given log *K*_ow_ value, assuming that small-sized, nonionized molecules exhibit maximal bioavailability and are not metabolized (Dimitrov et al. 2002, 2003). The baseline model was theoretically justified by a multicompartment diffusion model:


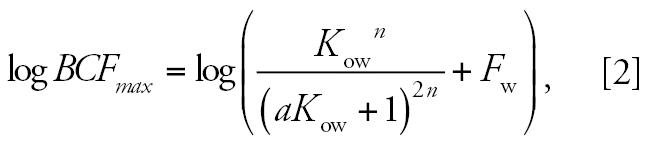


where *a* and *n* are fitted model parameters, and *F*_w_ is the water content of the organism.

Mitigating chemical properties (e.g., molecular size and flexibility, ionization, volatilization, adsorption) and organism specific properties (e.g., biotransformation, membrane permeability) are used as reducing factors of the maximum bioconcentration determined via the baseline model:


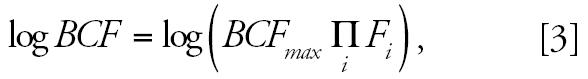


where *F*_i_ are the mitigating factors. Specific submodels have been developed for estimating *F*_metabolism_, F_ionization_, and F_molecular size_.

The POPs model parameters were optimized by making use of the training set of experimental BCF values for 542 chemicals. The model performance for the training set resulted in a correlation coefficient of *r*^2^ = 0.84, residual sum of squares = 140, and variance = 0.29. For 88% of the training set chemicals, the difference between observed and model-derived BCF values was within 0.75 log unit. In an external validation exercise with 176 chemicals similar to the model’s training set, the model demonstrated a similar predictability, with 80% of the BCF predictions falling within 0.75 log unit of the measured BCF value (Dimitrov et al. 2005b; Mekenyan et al. 2005).

The use of submodels for the mitigating chemical and organism properties has led to better estimation of measured BCF values (Dimitrov S, personal communication). An analysis of the relative importance of the three mitigating factors showed that passive diffusion limitations due to ionization and molecular size accounted for 69% of the model variance and metabolism 27%; all other mitigating factors accounted for the remaining 4% of model variance. A screening exercise recently performed on approximately 10,000 organic substances for Environment Canada revealed that, by incorporating all three submodels for mitigating factors in the POPs model, the number of chemicals identified as potentially bioaccumulative was reduced significantly (Dimitrov et al. 2002, 2003). Approximately 12.5% of the organic chemicals were identified as potentially bioaccumulative when only the submodel for molecular size was included as a mitigating factor for predicted BCF values, compared with 1.5% of the chemicals identified as potentially bioaccumulative when all the submodels for mitigating factors were used.

### Bioaccumulation models

Food web models can be used to predict BCF, BAF, and BSAF values for aquatic organisms; they are being used increasingly in PBT assessments because they incorporate dietary sources and other environmentally relevant processes that contribute to exposure. Since the 1970s, food web models have been created using data for POPs (Arnot and Gobas 2004; Hendriks et al. 2001; Morrison et al. 1997; Sharpe and Mackay 2000). Many of these chemicals are very slowly metabolized by aquatic species; this has enabled greater understanding of key bioavailability, uptake, and loss mechanisms in the environment. For substances that are subject to metabolic biotransformation, BAF values may be overpredicted if this loss rate is not included in the model’s parameters (Burkhard et al. 2003). Food web models have not been evaluated for reliability for all chemical classes (i.e., ionizing substances) because field bioaccumulation data are generally not available.

The application of a food web model requires the specification of the food web, ecosystem conditions (e.g., sediment–water column disequilibria of the chemical, organic carbon content of the sediment, dissolved and particulate organic carbon concentrations in water, average temperature), the biotransformation rates, and other related factors for all organisms of the food web (e.g., weights, lipid and water contents, prey species). Contaminant concentrations can vary widely among individual organisms in the environment, so population mean and variance are also important model parameters. The accuracy of the model is determined by comparing the model prediction (based on average values) to the means and SDs of measured BAF. Predicted BAF values from food web models can be reasonably accurate when they are properly constructed and when high-quality input data are used to make predictions. For example, one comparison of estimated and measured BAFs for multiple chemicals in fish from three ecosystems (*n* = 606) found 60% and 98% of the estimated log BAFs were within 0.3 and 1.0 log unit of their measured values, respectively, with a correlation coefficient (*r*^2^) of 0.88 (Arnot and Gobas 2004).

The application of typical food web models for screening large numbers of chemicals, such as for chemical management programs, is an arduous task because of the variability in site-specific ecosystem conditions and the input data required to simulate specific food webs. A semiempirical mass balance bioaccumulation model was developed to address these limitations, providing a generic site assessment method (Arnot and Gobas 2003). The model circumvents many of the required site-specific input parameters by calibrating BAF predictions to measured BAF data. The model delivers a BAF prediction for a selected general trophic level (e.g., lower, middle, upper), requiring only a *K*_ow_ value for the chemical. Calibrating the model to BAF data for poorly metabolized chemicals allows for estimates of food web bioaccumulation potential. If reliable metabolic biotransformation rate data are available, these can be included in the mass balance calculations. The model can also provide BCF estimates by excluding dietary uptake.

The growing interest in determining the ADME processes for chemicals in fish was the subject of the *In Vitro* ADME Workshop. Workshop participants explored the development and validation of techniques for extrapolating subcellular or *in vitro* measurements to whole body biotransformation rates or enzymatic activity rates across fish species that could then be used as stand-alone bioaccumulation assessments or incorporated into BCF and BAF model predictions.

## Conclusions

This report from the In Vivo Bioaccumulation Database Workshop is the first communication from a series of workshops organized to improve bioaccumulation assessment science. The purpose of the workshop was to identify the main sources of bioaccumulation data, to assess and understand existing empirical bioconcentration and bioaccumulation data, and to set a path to improve predictive models and their use. Empirical data were found in 12 databases from around the world (Table 1). When measured bioaccumulation data are unavailable, government agencies responsible for chemical management programs generally use four types of predictive computer models: the BCFWIN model, the POPs model, mass balance food web models, and *K*_ow_-based QSAR regressions.

The first significant finding of the bioaccumulation database workshop was that most data residing in the 12 databases are from the same studies or sources. Further, no one database appears to contain all of the bioaccumulation data that could be made public. Consequently, researchers, regulators, and modelers have to sort through a number of different data sources, with different formats and access methods, in order to assemble bioaccumulation data sets. This finding led to the recommendation that disparate sets of bioconcentration and bioaccumulation data should be combined into one easily accessible and assessable database. The development of a single metadatabase of all available bioaccumulation data would greatly simplify and improve data access and eliminate duplicate records of the same data, currently a difficult problem when combining data from different sources.

The second significant finding of the workshop was that different data sources contained different amounts of supporting information. Some sources simply provided a BCF, BAF, or BSAF value, whereas others provided additional supporting experimental information, such as fish species or water temperature. Only the Environment Canada database provides a reliability evaluation. This finding led to the second recommendation of the workshop: the reliability of existing data needs to be evaluated so that a “quality approved” set of data are available. Assembling data sets of known quality would enable better assessments of bioaccumulation as well as the development of improved predictive models.

The third significant finding of the workshop was that there is a need to move away from the use of the BCF alone, as well as the use of the *K*_ow_ to predict BCF, for assessing the bioaccumulation potential of organic chemicals. The use of the BCF alone ignores biomagnification and biotransformation processes in aquatic and terrestrial food webs.

## Figures and Tables

**Table 1:** Sources of bioconcentration and bioaccumulation data.

Database	Chemicals	Public access?
Japan METI-NITE Biodegradation and Bioconcentration	800 “existing”; ~ 3,100 “new”	Yes by Internet (NITE 2006) for “existing” substances only; data for “new” chemicals are proprietary and not available
ECOTOX	~ 700	Yes by Internet (U.S. EPA 2006a)
NLM Hazardous Substances	Hundreds	Yes by Internet (NLM 2006)
Environment Canada BCF and BAF	842	By request from Environment Canada, existing substances
RIVM	~ 700	No
SRC Fish BCF & Environmental Fate	694	Yes by Internet (SRC 2006)
CONCAWE (Hydrocarbons) BCF	84	No
U.S. Army ERDC BSAF	205	Yes by Internet (ERDC 2006)
U.S. EPA Superfund BSAF	In progress	Expected 2007
Handbook of Physical Chemical	Hundreds	Yes (see Mackay et al. 2006)
Properties and Environmental Fate
Comparative QSAR	209	Yes (see Devillers 1998)

Abbreviations: CONCAWE, Conservation of Clean Air and Water in Europe; ECOTOX, U.S. EPA ECOTOXicology; ERDC, Engineer Research and Development Center; METI-NITE, Ministry of Economy, Trade and Industry–National Institute of Technology and Evaluation; SRC, Syracuse Research Corporation.
